# Substrate stiffness regulates HIF-1α signalling via β1-integrin/myosin-2/NF-κB axis

**DOI:** 10.1186/s12967-026-08553-9

**Published:** 2026-07-09

**Authors:** Ander Bastida Urkiza, Ernesto Cortes, Chongguang Jin, Dariusz Lachowski

**Affiliations:** 1 Department of Neuroscience and Biomedical Sciences, Universidad Carlos III de Madrid, Madrid, 28903 Spain; 2 Department of Bioengineering, Imperial College London, London, SW7 2AZ United Kingdom; 3 Department of Physiology, School of Medicine, Autonomous University of Madrid, Madrid, 28029 Spain

**Keywords:** Hypoxia-inducible factor, Alpha subunit, Substrate stiffness, Transcriptional regulation, Cytoskeletal tension

## Abstract

**Background:**

HIF-1α is classically activated under hypoxia, inducing metabolic adaptation, angiogenesis, extracellular matrix remodelling and immune response. However, HIF-1α activation under normoxia and the role of mechanical stimuli on hypoxic response remain incompletely characterized. This study investigates the role of mechanical stimuli in modulating HIF-1α activation under normoxia by studying the response to matrix stiffness, and the role of NF-κB pathway in this modulation.

**Methods:**

PSCs were cultured on polyacrylamide hydrogels of defined stiffness to study HIF-1α modulation and its functional activation. The mechanosensitive regulation of NF-κB signalling, β1 integrin activation, and the RhoA-dependent MLC-2 pathway were studied. Pharmacological modulation of this mechanosensitive pathway was performed by targeting GPER as an upstream mechanoregulator.

**Results:**

Substrate stiffness induced robust nuclear translocation and functional activation of HIF-1α under normoxia. Mechanistically, this response required the β1 integrin-induced actomyosin contractility (mediated by MLC-2), promoting the activation of NF-κB, thereby positively regulating HIF-1α mRNA levels. The intermediate NF-κB activation was required to induce functional activation of HIF-1α, coupling mechanical stimuli to inflammatory and hypoxia signalling. This signalling pathway was targeted pharmacologically by regulating actomyosin tension using a GPER agonist, which modulates both mechanosensitive NF-κB and HIF-1α activation.

**Conclusions:**

These results reveal that substrate stiffness induces HIF-1α activation through the β1 integrin/MLC-2/NF-κB axis, implicating a mechanosensitive regulatory pathway under normoxia. These findings propose a novel feed-forward loop between matrix stiffness and HIF-1α signalling that can be pharmacologically modulated.

**Supplementary information:**

The online version contains supplementary material available at https://doi.org/10.1186/s12967-026-08553-9.

## Introduction

Hypoxia-inducible factor 1 (HIF-1) is a master transcriptional regulator composed of an oxygen-regulated HIF-1α subunit and a constitutive HIF-1β subunit [1]. HIF-1 plays a central role in maintaining cellular homeostasis. It orchestrates cellular and systemic adaptations to stress, particularly hypoxia, by controlling genes involved in metabolic reprogramming. HIF-1 achieves this by: increasing glycolysis regulation and lactate production [2]; inducing angiogenesis and vascular remodelling [3]; regulating cell survival and proliferation [4]; inducing extracellular matrix (ECM) remodelling, increasing ECM deposition and stiffness [5, 6]; and regulating immune response and inflammatory response [2]. HIF-1 is also a central regulator of cellular stress response under pathological conditions; for instance, it reprograms cancer metabolism in the tumour microenvironment (TME). In cancer, HIF-1α drives metabolic plasticity, angiogenesis and epithelial-mesenchymal transition (EMT) [7–9]; this correlates HIF-1α with increased tumour growth, immune evasion, and therapeutic resistance [4].

Canonically, HIF-1α is constitutively synthesized but under normoxia is rapidly hydroxylated (on amino acids Pro402 and Pro564) by oxygen-sensitive prolyl hydroxylase (PHD) enzymes, most notably PHD2. Hydroxylated HIF-1α is recognized and ubiquitinated by the von Hippel-Lindau (VHL) complex and degraded by proteasomal activity [10]. Under hypoxia PHD activity is inhibited, stabilizing HIF-1α, which dimerizes with HIF-1β and regulates target gene transcription by binding to hypoxia response elements (HREs) [10]. While hypoxia-driven HIF-1α stabilization is well established, increasing attention is directed towards HIF-1α activation under normoxia, known as pseudohypoxia [11]. Several mechanisms have been identified to activate HIF-1α under normoxia. Reactive oxygen species (ROS) have been shown to promote HIF-1α accumulation through the inhibition of PHD or the post-transcriptional stabilization of HIF-1α [12]. In addition to ROS-mediated regulation, recent experimental evidence demonstrates that tumour-derived metabolites can directly modulate HIF-1α stability. Lactate-driven post-translational lactylation of HIF-1α has been shown to impair VHL recognition and enhance HIF-1α stability independently of oxygen availability, providing biochemical evidence for metabolite-driven pseudohypoxic activation [13]. In cancer, HIF-1α activation under normoxia has been identified as a functionally significant phenomenon driven by oncogenic signalling. Both the PI3K/Akt/mTOR [14] and the MAPK/ERK [15] pathways can enhance HIF-1α by increasing its transcript levels to induce its functional activation. Beyond oncogenic signalling, recent work has further shown that chromatin-associated regulators integrate stress responses with HIF-1α-dependent metabolic reprogramming. For instance, the E3 ligase RNF20 was demonstrated to regulate HIF-1α stability and tumour metabolic rewiring in lung cancer, linking DNA damage responses to HIF-1α–mediated transcriptional programmes independently of canonical hypoxia signalling [16]. Inflammatory signalling also upregulates HIF-1α mRNA levels in response to cytokines, notably mediated by STAT3 or nuclear factor kappa B (NF-κB) activation [17, 18].

Inflammatory signalling represents a major axis through which HIF-1α activity under normoxia is regulated, with NF-κB serving as a key mediator of this activation. NF-κB is a central transcription factor controlling inflammatory, immune, and survival pathways [19]. Mechanistically, NF-κB transcriptionally regulates HIF-1α expression [18]. The interplay between NF-κB and HIF-1α activation is bidirectional under onset hypoxia, where NF-κB remains active, as stabilized HIF-1α further activates NF-κB signalling by inducing phosphorylation of IKKβ [20, 21], reinforcing inflammatory transcriptional programmes. This reciprocal crosstalk is particularly relevant within the TME, where inflammatory signals, hypoxia, and metabolic reprogramming converge to sustain tumour progression [22]. In this context, HIF-1α functions not only as a downstream effector of inflammatory signalling but also as a regulator of immune cell metabolism. Recent experimental studies demonstrate that sustained HIF-1α–dependent glycolysis supports effector T-cell function, highlighting its role in shaping immune responses independently of classical oxygen deprivation [23]. Thus, HIF-1α integrates inflammatory cues with metabolic programming to coordinate both tumour-intrinsic and immune cell adaptation within the TME. Importantly, the TME is not defined solely by biochemical and inflammatory signals but also by profound mechanical alterations, including increased matrix stiffness and cytoskeletal tension.

Emerging evidence suggests that mechanosensitive pathways play a key role on the regulation of NF-κB and HIF-1α stabilization in response to mechanical stress, indicating that biomechanical cues cooperate with inflammatory and metabolic signals to sustain HIF-1α activity under normoxic conditions. HIF-1α is stabilised and activated by compressive stress in periodontal ligament fibroblasts, mediated by PRK2/p-ERK activation [24]. HIF-1α is also activated by low shear stress in endothelial cells, mediated by the intermediate activation of NF-κB [25]. This study coupled for the first time mechanical stimuli to inflammatory and metabolic transcriptional programmes through the regulation of NF-κB and HIF-1α. Additional results indicating HIF-1α upregulation in response to increased substrate stiffness remained largely unexplored [26, 27] although the NF-κB activation in response to matrix stiffness was previously characterized [28, 29]. This interplay between substrate stiffness and HIF-1α activation still requires the characterization of the intermediate mechanosensitive signalling pathways. Integrins are primary mechanosensors linking the extracellular matrix to the cytoskeleton and conveying mechanical stimuli into biochemical signals [30]. Upon integrin activation, focal adhesion complexes are formed, leading to the activation of downstream signalling pathways, including focal adhesion kinase (FAK), Src family kinases, and Rho GTPases [30]. One of the main integrin activation-mediated downstream pathways is the induction of actomyosin contractility, whereby RhoA-mediated activation of ROCK induces myosin light chain 2 (MLC-2) phosphorylation, inducing actin filament tension, enabling cells to generate and transduce mechanical forces [31]. This integrin-driven coupling between focal adhesions, cytoskeletal dynamics and force generation constitutes a core mechanism mediating cellular mechanotransduction and thus a potential candidate for HIF-1α regulation.

Despite these advances, the mechanisms governing HIF-1α activation under normoxia and the potential role of mechanosensitive pathways remain to be defined. Elucidating how biomechanical and inflammatory pathways integrate to regulate HIF-1α activation is essential to understand tumour adaptation mechanisms and to identify potential novel therapeutic approaches. Here, we investigate the regulation and functional activation of HIF-1α under normoxia in response to mechanical stimuli, focusing on its interplay with the NF-κB pathway. By identifying these mechanosensitive pathways, we provide mechanistic insight into stress-responsive transcriptional networks in response to mechanical stimuli.

## Materials and methods

### Cell culture and experimental design

Human pancreatic stellate cells (PSCs, HPaSteC, #3830, ScienCell, CA, USA) or GE11 cells were cultured in DMEM/F-12 (D8437, Sigma Aldrich) or Dulbecco’s modified Eagle’s media (D6429, Sigma Aldrich) respectively, supplemented with 10% v/v FBS (10270106, Gibco), 1% v/v Penicillin-Streptomycin (P4333, Sigma-Aldrich) and 1% v/v Amphotericin B (15290026, Gibco) under standard conditions (37 °C, 5% CO_2_).

Pharmacological inhibitors and functional blocking antibodies were used when indicated in individual experiments. HIF-1α degradation was inhibited with DMOG (sc-20075, Santa Cruz Biotechnology) 100 µM for 96 h, media was refreshed after 48 h. Functional blocking was performed using anti-β1 integrin (Cat. No. 552828, BD Biosciences) 10 µg/mL, or anti-αVβ3 integrin (Cat. No. MAB1976Z, Millipore) 10 µg/mL antibodies respectively. Actomyosin contractility was inhibited using either RhoA inhibitor CT04 (CT04, Cytoskeleton) 2 µg/mL for 4 h, or MLC-2 inhibitor Blebbistatin (HY-13813, MedChemExpress) 50 µM for 4 h. For all conditions involving inhibition of actomyosin contractility, culture medium was replaced with serum-free medium for the duration of drug treatment. GPER was pharmacologically targeted with agonist G-1 (3577, Tocris) 1 µm for 72 h in complete media.

Cells were transfected with 15 pmol NF-κB siRNA (sc-29410, Santa Cruz Biotechnology) or control scr siRNA (sc-37007, Santa Cruz Biotechnology) using Lipofectamine 3000 (L3000001, Invitrogen) according to the manufacturer’s protocol. Downstream assays were performed 24 h post-transfection.

### Polyacrylamide substrates

To investigate the effect of substrate stiffness, cells were cultured on polyacrylamide (PAA) hydrogels of defined stiffness when indicated. PAA hydrogels were prepared as previously described [27] adjusting acrylamide/bis-acrylamide (29:1) (A7802, Sigma-Aldrich) concentration to obtain substrates of low (1.3 kPa) and high (25.5 kPa) stiffness, following the formulation given in Table 1. In short, glass coverslips were pre-treated with 3-(trimethoxysilyl)propyl methacrylate (440159, Sigma-Aldrich) and placed covering a 30 µL drop of the prepolymer mixture. After PAA polymerization hydrogels were functionalized using Sulfo-SANPAH (803332, Sigma-Aldrich), and coated with fibronectin (RP-43130, Invitrogen) at 10 μg/mL in PBS for 16 h at 4 °C. Cells were cultured at a density of 20.000 cells/cm^2^ and allowed to adhere for 24 h before downstream assays.

**Table 1:** Acrylamide/bis-acrylamide concentrations and desired PAA stiffness

Stiffness (kPa)	PBS (µL)	Acrylamide/bis-acrylamide [29:1] 40% vol (µL)	TEMED (µL)	10% APS (µL)
1.3	459.1	34.9	1	2.5
25.5	378.7	125.3	1	2.5

### Immunofluorescence imaging

Cells were fixed with 4% w/v paraformaldehyde (P6148, Sigma Aldrich) in PBS for 10 min, permeabilized for 10 min with either 0.5% w/v saponin (47036, Sigma Aldrich) in PBS for cytoskeletal proteins or 0.1% v/v Triton X-100 (X100, Sigma Aldrich) in PBS for nuclear factors, and blocked with 1% w/v BSA (A8022 Sigma Aldrich), 0.1% Tween-20 (P6585, Sigma Aldrich), 22.52 mg/mL glycine (G8898, Sigma Aldrich) in PBS for 30 min. After blocking, cells were incubated with primary antibodies with 1% w/v BSA, 0.1% Tween-20 in PBS overnight at 4 °C in a humidified chamber. Cells were stained with conjugated secondary antibodies and Alexa Fluor 546 phalloidin (A22283, Invitrogen, 1:500) with 1% w/v BSA in PBS for 1 h at room temperature and mounted with ProLong Gold Antifade Mounting medium containing DAPI (P36934, Invitrogen).

The antibodies used were anti-HIF-1α antibody (ab179483, abcam, 1:200); anti-NF-κB p65 monoclonal antibody (#8242, Cell Signaling Technology, 1:100); anti-integrin β1 antibody, activated (MAB2079Z, Sigma Aldrich, 1:200); anti-alpha smooth muscle actin antibody (ab124964, abcam, 1:300); anti-vimentin antibody (ab92547, abcam, 1:200); goat anti-rabbit IgG (H+L) Alexa Fluor 488 (A11034, Invitrogen, 1:200); and goat anti-mouse IgG (H+L) Alexa Fluor 488 (A11029, Invitrogen, 1:200).

Widefield and confocal images were acquired with a Nikon Ti-e Inverted Microscope (Ti Eclipse, CFI Apo TIRF 60X/1.49 oil objective, CFI Plan Fluor 40x NA 0.6 air objective) and the NIS elements AR software; or with a Zeiss LSM 700 Microscope (Plan-Apochromat 40x/1,40 Oil DIC objective) and the ZEN 2011 software. For total internal reflection fluorescence (TIRF) microscopy, cells were cultured in confocal dishes coated with either human vitronectin (CC080, Sigma Aldrich) at 2 µg/mL in PBS, or collagen type I (354249, Corning) at 50 µg/mL in 0.02 M acetic acid. TIRF images were acquired with a Nikon Ti-e Inverted Microscope (Ti Eclipse, CFI Apo TIRF 60X/1.49 oil objective) operating in TIRF mode.

### Luciferase activity

Cells were transfected with 500 ng HRE-luciferase (#26731, addgene) cDNA using Lipofectamine 3000 (L3000001, Invitrogen) according to the manufacturer’s protocol. Following culture on substrates of defined stiffness or pharmacological treatment, luciferase activity was measured using Luciferase Assay System (E4030, Promega) according to the manufacturer’s protocol.

### RT-qPCR

Total RNA was isolated using the RNeasy Mini Kit (74104, Qiagen), and reverse-transcribed using the High-Capacity RNA-to-cDNA kit (4387406, Applied Biosystems). Gene expression of HIF-1α was measured using SYBR Green Universal Master Mix (4309155, Applied Biosystems). Expression levels were normalized to RPLP0 and calculated using the ΔΔCt method. Primer sequences used were: HIF1A (F) 5’-AAAATCTCATCCAAGAAGCC-3’, (R) 5’-AATGTTCCAATTCCTACTGC-3’; LOX-L2 (F) 5’-GATGTACAACTGCCACATAG-3’; (R) 5’-GACAGCTGGTTGTTTAAGAG-3’; LOX-L3 (F) 5’-AGGAAACTACATTCTCCAGG-3’; (R) 5’-ACCCAGATTCTATGTCCATC-3’; RPLP0 (F) 5’-CGGTTTCTGATTGGCTAC-3’, (R) 5’-ACGATGTCACTTCCACG-3’.

### Statistical analysis

Data are presented as mean ± SEM unless otherwise indicated. All statistics were conducted using GraphPad Prism (version 9, GraphPad). For comparisons between two groups, a two-tailed Student’s t-test was used for normally distributed data, while the Mann-Whitney U test was applied for non-parametric samples. For multiple comparisons, one-way ANOVA followed by Tukey’s or Dunnett’s post hoc tests were used for normally distributed data; non-normally distributed data were analysed using the Kruskal-Wallis test followed by Dunn’s post hoc multiple comparison test. Significance was set at *p* < 0.05, statistical significance is indicated by: * 0.05 > *p* ≥ 0.01, ** 0.01 > *p* ≥ 0.001, *** 0.001 > *p* ≥ 0.0001, **** 0.0001 > p.

## Results

### Substrate stiffness upregulates HIF-1α activation under normoxia

Our previous work suggests that HIF-1α may be activated in response to substrate stiffness under normoxia, driven by mechanical stimuli that bypass the canonical oxygen-dependent HIF-1α regulation [26, 27]. The potential implication for disease pathology and therapeutic response prompted us to investigate the effect of mechanical stimuli on HIF-1α functional activation under normoxia. Pancreatic stellate cells (PSCs) are the main stromal effectors in response to mechanical stress in the pancreas and the dominant source of ECM deposition in tumour desmoplasia. In pancreatic ductal adenocarcinoma (PDAC), matrix stiffness induces PSC activation, assuming a myofibroblast-like morphology. PSC activation amplifies oncogenic signalling and establishes a feed-forward fibrotic microenvironment [32–34]. PSCs provide a physiologically relevant and mechanistically robust model to investigate HIF-1α activation in cancer and fibrosis. To address this question, we investigated HIF-1α activation in primary PSCs cultured on fibronectin-coated polyacrylamide (PAA) substrates of defined stiffness under normoxia. As validated in previous pancreatic mechanobiology models soft (1 kPa) and stiff (25 kPa) substrates were used to recapitulate mechanical properties of healthy and highly fibrotic pancreas, respectively [35].

**Fig. 1:**
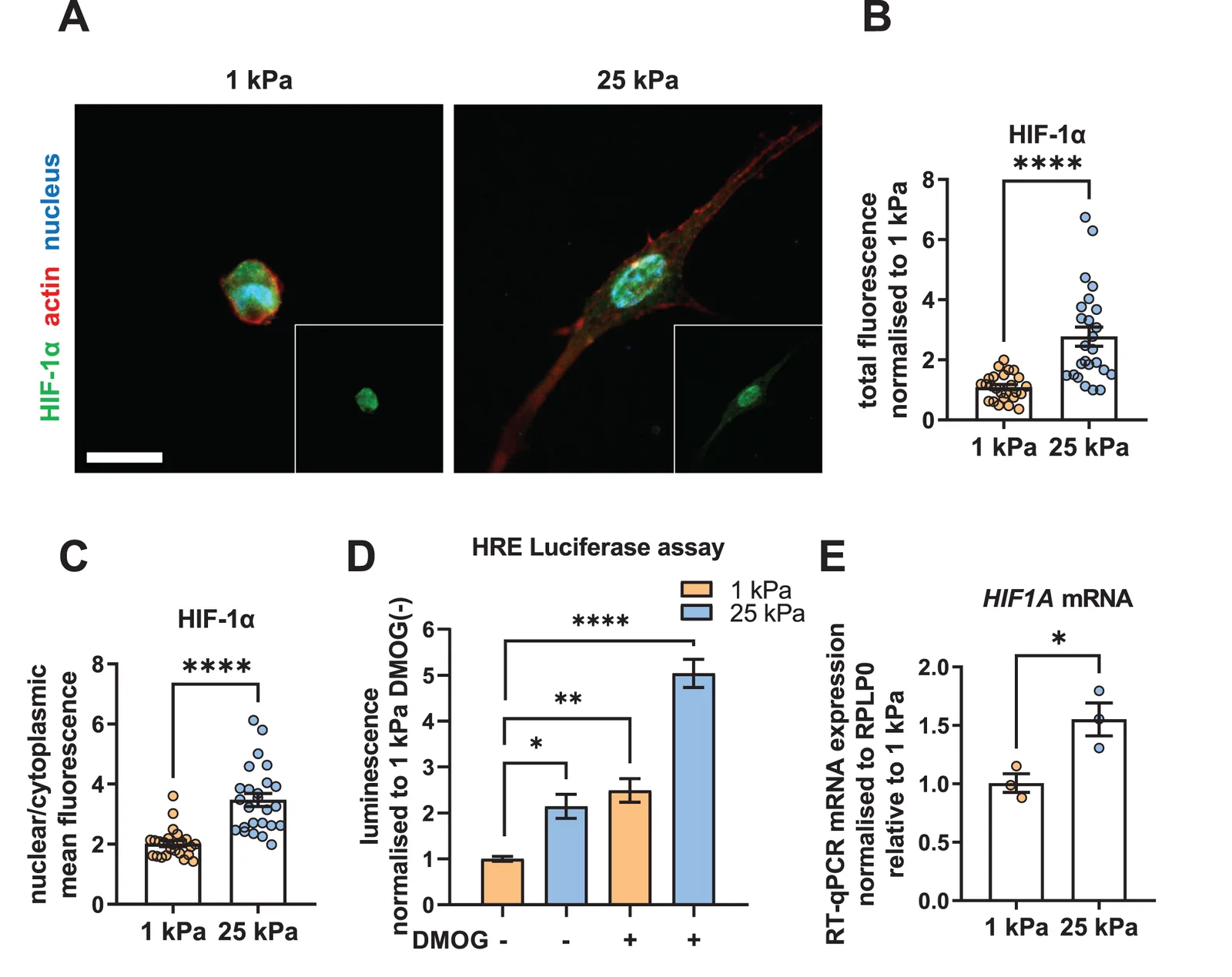
Substrate stiffness modulates HIF-1α nuclear translocation and transcriptional activation. (**A**) Widefield immunofluorescence images of PSCs for HIF-1α cultured on soft (1 kPa) or stiff (25 kPa) substrates. Scale bar, 20 µm. (**B**) Total fluorescence intensity of HIF-1α per cell, as shown in (**A**) (*n* = 25 cells). (**C**) Mean HIF-1α fluorescence nuclear to cytoplasmic ratio per cell, as shown in (**A**) (*n* = 25 cells). (**D**) Relative luciferase activity of hypoxia response elements (HREs) of PSCs cultured on soft (1 kPa) or stiff (25 kPa) substrates, with dimethyloxallyl glycine (DMOG; 100 µM, 96 h) (*n* = 3 biological replicates). (**E**) Reverse transcription quantitative PCR detection of *HIF1A* gene in PSCs cultured on soft (1 kPa) or stiff (25 kPa) substrates (*n* = 3 biological replicates). Histograms represent mean ± SEM

We observed that increased substrate stiffness induced a marked increase in both the HIF-1α expression and in the HIF-1α nuclear translocation (Fig. 1A–C). To determine whether this nuclear translocation was accompanied by an increased transcriptional activity, HIF-1α functional activity was assessed by hypoxia response element (HRE)-luciferase [36]. Consistent with nuclear translocation, cells cultured in higher substrate stiffness showed a marked increase in luciferase activity (Fig. 1D). Interestingly, this increased functional activity was observed to be substrate stiffness-dependent even after PHD enzymes inhibition using dimethyloxalylglycine (DMOG). To further validate the substrate stiffness-dependent HIF-1α transcriptional activation the mRNA expression of classical HIF-1α target genes LOXL2 and LOXL3 was analysed. Consistent with luciferase activity the mRNA expression of these HIF-1α target genes was upregulated by substrate stiffness (Supplementary Figure S1). These results confirmed that substrate stiffness not only increases HIF-1α expression and nuclear translocation, but it further highlights that substrate stiffness can modulate HIF-1α transcriptional activation independent of its proteasomal degradation. The increased HIF-1α activation was also accompanied by a significant increase in HIF-1α mRNA expression, as measured by qPCR (Fig. 1E). These results further suggest that the elevated protein levels are primarily driven by increased synthesis rather than reduced HIF-1α degradation. Collectively, these findings demonstrate that PSCs adopt pathways characteristic of the hypoxic response induced by substrate stiffness under normoxia.

### Substrate stiffness-dependent HIF-1α activation is mediated by the NF-κB pathway

**Fig. 2:**
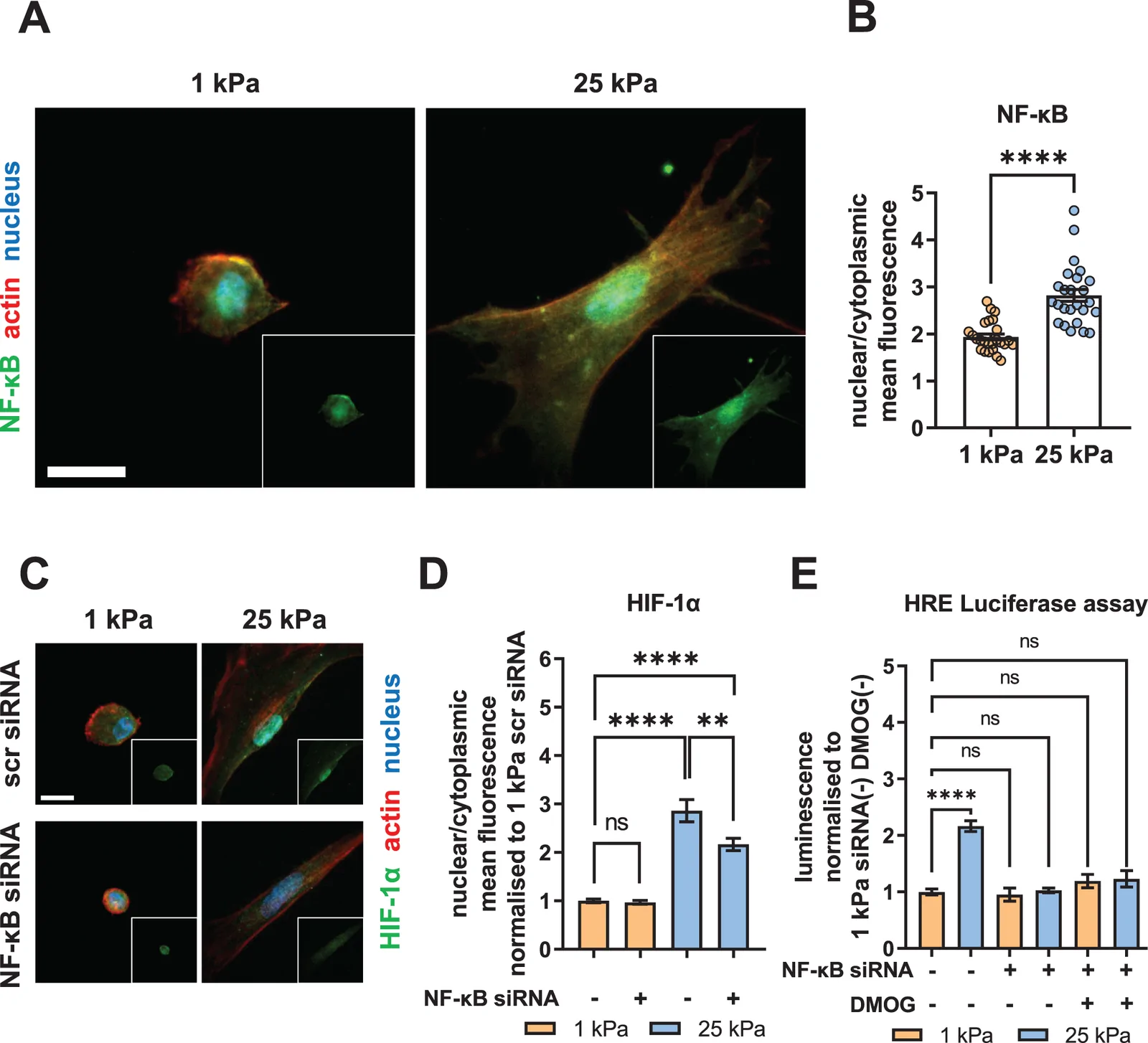
The NF-κB pathway mediates the mechanosensitive HIF-1α activation. (**A**) Widefield immunofluorescence images of PSCs for NF-κB cultured on soft (1 kPa) or stiff (25 kPa) substrates. Scale bar, 20 µm. (**B**) Mean NF-κB fluorescence nuclear to cytoplasmic ratio per cell, as shown in (**A**) (*n* = 25 cells). (**C**) Widefield immunofluorescence images of PSCs for HIF-1α cultured on soft (1 kPa) or stiff (25 kPa) substrates, transfected with NF-κB siRNA. Control scramble siRNA (scr siRNA). Scale bar, 20 µm. (**D**) Mean HIF-1α fluorescence nuclear to cytoplasmic ratio per cell, as shown in (**C**) (*n* = 35 cells). (**E**) Relative luciferase activity of hypoxia response elements (HREs) of PSCs cultured on soft (1 kPa) or stiff (25 kPa) substrates, transfected with NF-κB siRNA (control scr siRNA), and with dimethyloxallyl glycine (DMOG; 100 µM, 96 h) (*n* = 3 biological replicates). Histograms represent mean ± SEM

Our findings indicate that increased HIF-1α activity is driven by transcriptional upregulation rather than impaired proteasomal degradation. Consequently, we sought to elucidate the underlying mechanism responsible for this transcriptional activation. NF-κB is a central transcriptional regulator of inflammation and stress-induced signalling pathways. HIF-1α is a NF-κB target gene: the direct transcriptional regulation of HIF-1α by NF-κB is previously demonstrated both through ChIP and RT-qPCR [18]. The established mechanosensitive activation of NF-κB highlights it as a potential mediator of the observed mechanosensitive HIF-1α activation [18, 28]. We noted that similar to HIF-1α, increased substrate stiffness induced nuclear translocation of NF-κB (subunit p65) (Fig. 2A–B). The NF-κB co-activation follows a kinetic profile parallel to HIF-1α, which suggests both transcription factors may be part of a coordinated mechanosensitive signalling pathway.

To establish whether NF-κB acts as the upstream HIF-1α regulator, NF-κB expression was silenced by siRNA transfection. NF-κB silencing reduced the nuclear translocation of HIF-1α in response to increased substrate stiffness (Fig. 2C–D). However, the most pronounced effect of NF-κB silencing was observed for HIF-1α transcriptional activation regulation. HRE-luciferase activity, indicative of HIF-1α functional activation, was significantly reduced following NF-κB silencing (Fig. 2E). To exclude the downstream effects of NF-κB silencing being secondary to impaired PSC contractile polarization, we confirmed that substrate stiffness-induced alpha-smooth muscle actin (αSMA) upregulation was unchanged following NF-κB silencing or HIF-1α induction (Supplementary Figure S2).

These results show that NF-κB is necessary for the mechanosensitive functional activation of HIF-1α. It is further observed that inhibition of proteasomal HIF-1α degradation by DMOG was not sufficient to increase the transcriptional activity of HIF-1α in the absence of NF-κB (Fig. 2E). The observed partial uncoupling between HIF-1α nuclear translocation and its functional activity suggests that NF-κB may have a direct functional role on HIF-1α under normoxia. The potential functional role of NF-κB in HIF-1α activation is further supported by emerging evidence that indicates NF-κB functions as a critical co-factor in hypoxia-induced transcription. Under hypoxia, HIF-1α activation requires the cooperation of NF-κB to facilitate the activation of a substantial subset of hypoxia-responsive genes [37]. These recent observations expand on the canonical view of hypoxic gene regulation beyond HIF-1α-centric models. Our results here identify that NF-κB is a necessary mediator in the mechanosensitive HIF-1α activation. We hypothesize this activation may require a dual role of NF-κB: the NF-κB-induced HIF-1α mRNA upregulation, and a coordinated transcriptional programme between NF-κB and HIF-1α to integrate mechanical stimuli with hypoxic response for PSCs.

### β1 integrin activation modulates HIF-1α via RhoA activation-induced actomyosin contraction

**Fig. 3:**
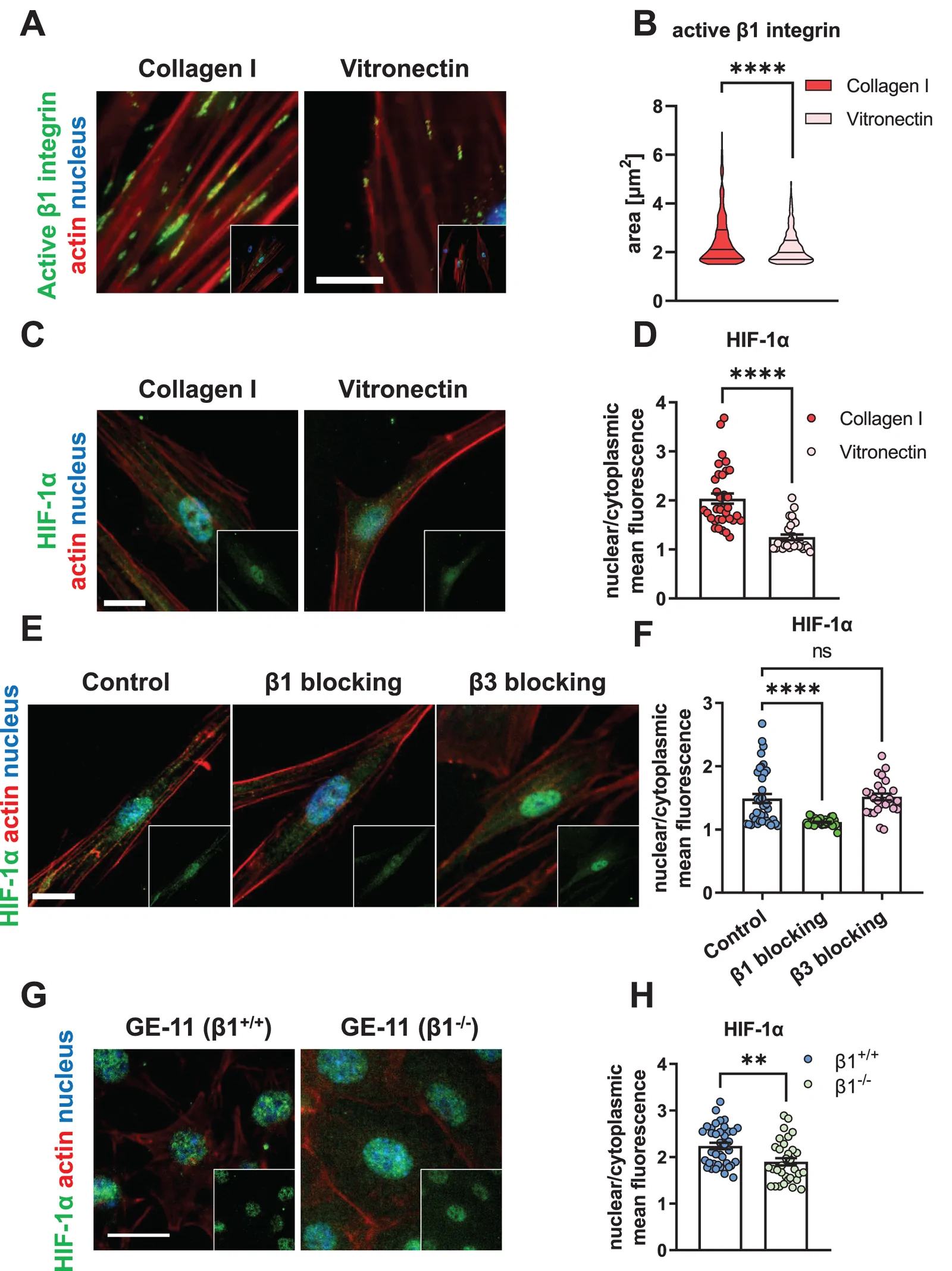
β1 integrin is the primary mechanosensor for substrate stiffness-dependent HIF-1α upregulation. (**A**) TIRF microscopy images of active β1 integrin (green) in the basal plane of PSCs cultured on collagen type I (collagen I) or vitronectin coated confocal dishes. Scale bar, 10 µm. (**B**) Active β1 integrin area per focal adhesion point, as shown in (**A**) (puncta from *n* = 25 individual cells). (**C**) Widefield immunofluorescence images of PSCs for HIF-1α cultured on collagen type I (collagen I) or vitronectin coated glass slides. Scale bar, 20 µm. (**D**) Mean HIF-1α fluorescence nuclear to cytoplasmic ratio per cell, as shown in (**C**) (*n* = 25 cells). (**E**) Widefield immunofluorescence images of PSCs for HIF-1α with and without β1 integrin or β3 integrin functional blocking. (**F**) Mean HIF-1α fluorescence nuclear to cytoplasmic ratio per cell, as shown in (**E**) (*n* = 25 cells). (**G**) Widefield immunofluorescence images of β1^-/-^ integrin (β1 integrin knockout) or β1^+/+^ integrin (β1 integrin overexpressing) mouse epithelioid GE11 cells. Scale bar, 20 µm. (**H**) Mean HIF-1α fluorescence nuclear to cytoplasmic ratio per cell, as shown in (**G**) (*n* = 25 cells). Violin plots indicate median and quartiles, histograms represent mean ± SEM

To determine the upstream mechanosensor driving the mechanosensitive HIF-1α activation, we first examined integrin activation on defined substrates. Active conformation β1 integrin staining exhibited an increase in β1 integrin puncta in PSCs cultured on collagen I relative to cells cultured on vitronectin (Fig. 3A–B). These results are consistent with the preferential β1 integrin engagement to collagen I compared to adhesion on vitronectin, which preferentially engages with alternative integrin subunits such as αVβ3 integrin. On these substrates HIF-1α activation was studied, observing a marked increase in the HIF-1α nuclear translocation for cells cultured on collagen I (Fig. 3C–D). These data suggest that the mechanosensitive HIF-1α activation observed on collagen I is potentially mediated through β1 integrin-dependent pathways.

To establish the β1 integrin-dependency for the mechanosensitive HIF-1α activation functional blocking antibodies for β1 integrin and αVβ3 integrin respectively were used. Following β1 integrin functional blocking we observed a significant decrease of HIF-1α nuclear translocation, not observed after αVβ3 integrin blocking (Fig. 3E–F). These results altogether indicate that the upstream integrin subunit activation is not redundant, and the specific requirement of β1 integrin activation for the present downstream HIF-1α activation.

To further confirm the role of β1 integrin in the proposed signalling pathway, we used β1 integrin knockout (β1^-/-^) or β1 integrin overexpressing (β1^+/+^) GE11 mouse epithelioid cells [38]. Consistent with the previous results a decrease in HIF-1α nuclear translocation in β1 integrin knockout cells was observed (Fig. 3G–H). Altogether, these results identify the β1 integrin subunit as the upstream mechanosensor conveying mechanical stimuli into the increased HIF-1α activation under normoxia.

Having identified β1 integrin as the upstream mechanosensor mediating HIF-1α activation, we next studied the downstream cytoskeletal pathways that transduce β1 integrin activation into functional responses. β1 integrin is a well-established activator of RhoA, a small Rho GTPase governing actomyosin contractility and regulating cellular tension [39, 40]. Given this canonical pathway, we hypothesize that substrate stiffness-induced β1 integrin activation and the RhoA-dependent actomyosin contraction may coordinate the HIF-1α upregulation under normoxia.

**Fig. 4:**
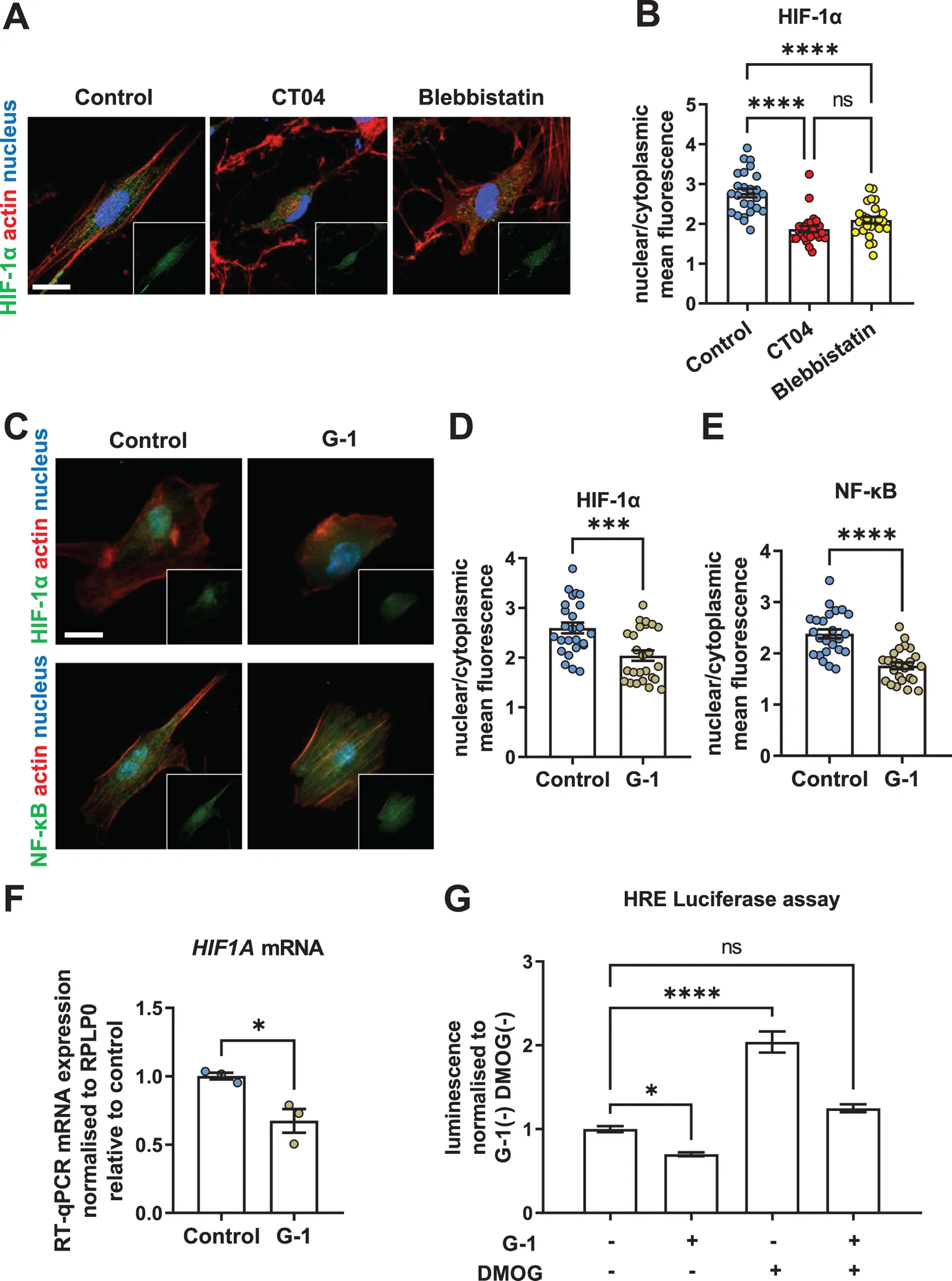
Actomyosin contractility is required for HIF-1α activation and can be pharmacologically modulated. (**A**) Confocal immunofluorescence images of PSCs for HIF-1α, treated with and without RhoA inhibitor (CT04; 2.0 µg/ml, 4 h) or myosin light chain 2 (MLC-2) inhibitor (Blebbistatin; 50 µM, 4 h). (**B**) Mean HIF-1α fluorescence nuclear to cytoplasmic ratio per cell, as shown in (**A**) (*n* = 25 cells). (**C**) Widefield immunofluorescence images of PSCs for HIF-1α and NF-κB, with and without GPER agonist (G-1; 1 µM, 72 h). (**D**) Mean HIF-1α (*n* = 25 cells) and (**E**) NF-κB fluorescence nuclear to cytoplasmic ratio per cell, as shown in (**C**) (*n* = 25 cells). (**F**) Reverse transcription quantitative PCR detection of *HIF1A* gene in PSCs treated with GPER agonist (G-1; 1 µM, 72 h) (*n* = 3 biological replicates). (**G**) Relative luciferase activity of hypoxia response elements (HREs) of PSCs treated with and without dimethyloxallyl glycine (DMOG; 100 µM, 96 h) or GPER agonist (G-1; 1 µM, 72 h) (*n* = 3 biological replicates). Histograms represent mean ± SEM

We studied HIF-1α activation following RhoA inhibition by CT04, or MLC-2 inhibition by Blebbistatin. We observe that either RhoA or MLC-2 inhibition markedly reduce the HIF-1α nuclear translocation (Fig. 4A–B). The concurrent reduction of HIF-1α nuclear translocation upon inhibition of both RhoA and MLC-2 reinforces a shared mechanotransducive axis, indicating that actomyosin contractility is required to convey β1 integrin-mediated mechanical stimuli into HIF-1α activation. Altogether, the proposed β1 integrin-dependent and NF-κB-mediated HIF-1α upregulation is consistent with the previously reported mechanosensitive NF-κB activation, dependent on the β1 integrin/RhoA/MLC-2 axis [28].

We next sought to determine whether this mechanosensitive HIF-1α activation could be pharmacologically modulated. Having identified the dependency on actomyosin contractility, we sought to modulate this mechanosensitive pathway in a manner that reduces contractility without directly disrupting the cytoskeleton. To this end, we propose G-1, a selective G protein-coupled oestrogen receptor (GPER) agonist that we previously identified in regulating cytoskeletal tension in PSCs [41]. GPER activation induces the Gs protein-mediated generation of cAMP. This generation activates two parallel pathways: PKA activation; and Epac/Rap1 activation. These two pathways inhibit the activation of RhoA and its effector ROCK, leading to decreased MLC-2 phosphorylation [42]. GPER activation by G-1 effectively inhibits RhoA activation, MLC-2 phosphorylation and reduces contractile force generation [42–44]. To evaluate the pharmacological advantages of G-1, we evaluated its effects with the direct RhoA and MLC-2 inhibitors. The direct RhoA and MLC-2 inhibitors (CT04 and Blebbistatin) caused pronounced cytoskeletal collapse and loss of structural organization as observed by increased actin cytoskeleton branching and loss of directional organization (Fig. 4A, Supplementary Figure S3), whereas G-1-treated cells preserved their actin architecture despite exhibiting reduced mechanosensitive induction (Supplementary Figure S4A-F). These results show that targeting GPER reduces PSC contractile polarization, observed by decreased αSMA and vimentin expression (Supplementary Figure S4G-I), through a less disruptive, more physiologically aligned means of attenuating actomyosin-dependent processes.

**Fig. 5:**
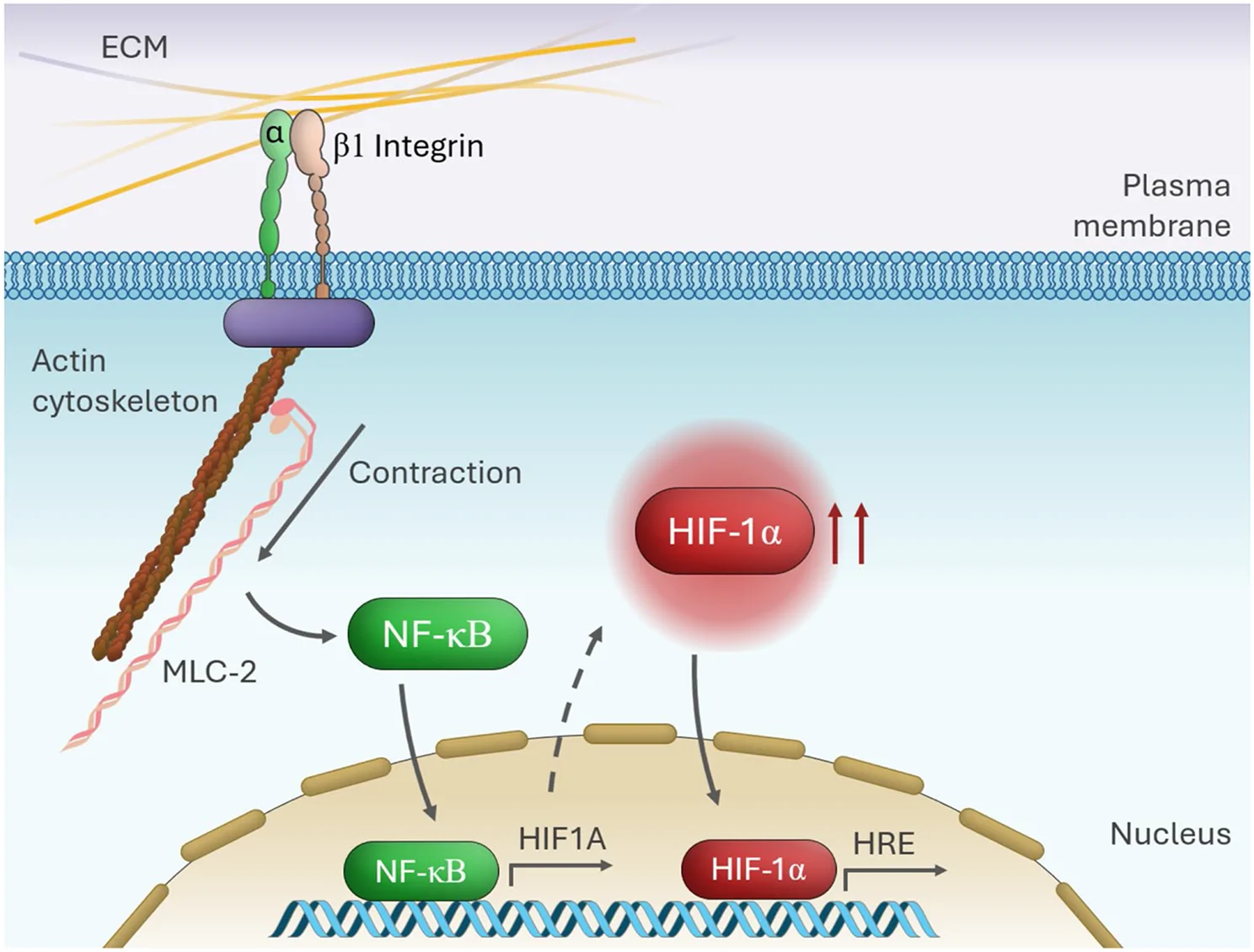
Schematic illustrating the mechanosensitive HIF-1α activation pathway in normoxia

To evaluate translational relevance, we next studied the effects of targeting GPER as upstream mechanosensor on regulating the RhoA-dependent mechanosensitive pathway. G-1 treatment significantly reduced HIF-1α nuclear translocation (Fig. 4C–D), paralleled by the reduction of NF-κB nuclear translocation (Fig. 4E). G-1 treatment modulated transcriptional activity by decreasing HIF-1α mRNA levels (consistent with NF-κB downregulation) (Fig. 4F) and a marked decrease in HRE-luciferase activity (Fig. 4G). We observed that G-1 treatment reduced the HIF-1α luciferase activity even in the presence of DMOG, consistent with the effect observed in response to differential substrate stiffness. These results confirm that the mechanosensitive HIF-1α modulation is highly sensitive to pharmacological modulation, as G-1 effectively modulates the coordinated transcriptional programme between NF-κB and HIF-1α. Altogether, these results establish the β1 integrin/RhoA/MLC-2/NF-κB axis as mediator of the mechanosensitive HIF-1α activation (Fig. 5); while also establishing GPER as a targeted, upstream modulator of this mechanosensitive pathway.

## Discussion

Previous studies have shown that HIF-1α activation can be induced in response to various stressors under normoxia, expanding HIF-1α relevance beyond its canonical role as a metabolic regulator under hypoxia. In this study we demonstrate that HIF-1α can be activated in response to substrate stiffness under normoxia. We identify NF-κB as a necessary mediator, induced by upstream β1 integrin/MLC-2, to induce HIF-1α transcriptional activation. Although mechanical stimuli and HIF-1α activation have been previously correlated [25, 27], here we propose a mechanosensitive signalling axis as mediator of this HIF-1α activation. These findings position HIF-1α as a broader stress-responsive transcriptional factor, supporting a model in which mechanical stress constitutes a potent, oxygen-independent regulator of hypoxia signalling.

Early evidence linking mechanical stress to HIF-1α was reported in the vascular endothelium, first proposing a mechanosensitive pathway. Low shear stress was shown to induce NF-κB activation and, in turn, transcriptionally upregulate HIF-1α under normoxia [25]. These findings provided a conceptual framework for oxygen-independent regulation of hypoxia signalling through mechanically induced inflammatory pathways. Extending beyond the vascular compartment, mechanosensitive activation of NF-κB has been reported in other cell lines, reinforcing its role as a central transducer of mechanical stress [28]. While this mechanosensitive NF-κB activation has been extensively studied, its potential involvement on hypoxia signalling has not been completely characterized, and the underlying pathways remained unexplored. Our data defines NF-κB as necessary signalling intermediary link between mechanical cues to hypoxia responses, integrating previously disconnected observations and providing a mechanistic basis for mechanosensitive, oxygen-independent HIF-1α signalling.

This oxygen-independent HIF-1α activation is likely to represent a dominant signalling pathway in ECM-rich solid tumours, where desmoplasia and elevated interstitial pressure induce a persistent mechanical stress [45]. This is particularly relevant to better understand metabolic plasticity at the TME, as mechanical alterations at the TME often precede the development of hypoxia during tumour evolution [46]. HIF-1α activation in pseudohypoxic conditions has profound effects on tissue remodelling. At atheroprone regions stress-dependent HIF-1α stabilization has functional implications for tissue remodelling. The mechanosensitive HIF-1α activation induces endothelial inflammation and vascular dysfunction via modulation of endothelial cell metabolism [25]. Cancer-associated fibroblast (CAF)-rich tumour stroma and the tumour invasive fronts also correlate ECM and HIF-1α activity [46], where HIF-regulated collagen deposition and LOX/LOXL upregulation is observed [47].

These previous works stress the role of HIF-1α activation on promoting tissue remodelling, highlighting that HIF-1α transcriptionally modulates ECM remodelling at various stages. HIF-1α upregulates collagen-modifying enzymes (P4HA1/2), lysyl hydroxylase PLOD2, and LOX/LOXL crosslinkers [48, 49]. These factors contribute to the deposition, fibril maturation and alignment of ECM components. HIF-1α also upregulates MMP secretion, further contributing to ECM remodelling [50, 51]. This dynamic ECM induces CAFs to secrete growth factors (TGF-β) and angiogenic factors release (VEGF), further contributing to TME remodelling [52, 53]. These previous studies couple tissue mechanics and ECM deposition to HIF-1α signalling at the TME, while our work establishes the influence of matrix stiffness on inducing HIF-1α activation. In ECM-rich solid tumours, mechanosensitive activation of HIF-1α may therefore directly promote further matrix deposition and stiffening, establishing a feed-forward loop in which ECM remodelling amplifies mechanical stress and sustains HIF-1α signalling. This observation has important implications for spatial models of HIF-1α activity within tumours. Rather than HIF-1α being only active at hypoxic tumour cores, HIF-1α activation may therefore represent an early and sustained driver of tumour adaptation and evolution, operating in parallel with classical hypoxia-driven signalling.

The role of HIF-1α activation in remodelling the stiff, collagen-rich TME has major consequences to cancer progression. Both mechanical stress and HIF-1α signalling induce cancer cell motility and invasion, being a determinant factor for metastatic competence [54–56]. The increased stiffness and interstitial pressure further contribute to cancer development by impairing immune cell infiltration into the tumour core [57, 58]. Additionally, HIF-1α induces chemoresistance in solid tumours by contributing to tumour metabolic adaptation and by the positive transcriptional regulation of cell survival pathways [59]. The recognition of mechanosensitive, normoxic HIF-1α activation may therefore have important implications for the understanding and treatment of solid tumours. Current HIF-1α-targeted therapies demonstrating preclinical antitumour activity include several strategies: small-molecule inhibitors impairing HIF-1α transcription, translation or dimerization; hypoxia-activated prodrugs; and upstream pathway modulators, such as PI3K/mTOR inhibitors [60–64]. However, no HIF-1α-centric agent has achieved regulatory approval as a monotherapy, as clinical effectiveness has been limited by limited responses, impaired delivery into the tumour, and toxicity concerns [64, 65]. Biological redundancy, adaptive resistance and the influence of pseudohypoxic signalling limit HIF-1α-targeted therapy efficacy, highlighting the necessity to address non-canonical pathways influencing HIF-1α activation [66]. Although it has been previously reported that tamoxifen, a GPER agonist, could influence HIF-1α regulation under normoxia, these observations remained strictly correlative, and the underlying signalling pathway remained unknown [26]. Our results could clarify the basis of the previously unexplained mechanical effects attributed to tamoxifen. By demonstrating that GPER activation using G-1 inhibits the mechanosensitive HIF-1α activation through RhoA regulation, we provide a mechanistic link between tamoxifen treatment and HIF-1α modulation. Tamoxifen treatment demonstrated effective joined regulation of HIF-1α activation and ECM remodelling, followed by decreased collagen synthesis, deposition and alignment [26]. Targeting upstream mechanosensitive pathways or cytoskeletal tension may therefore represent complementary strategies to modulate HIF-1α activity in solid tumours. The results here support the pharmacological potential for targeting upstream mechanosensitive modulators, demonstrating translational relevance. Such approaches may be particularly relevant for highly desmoplastic cancers where mechanical stress is a defining feature of the disease, as for breast cancer or pancreatic cancer.

While this study establishes a clear link between mechanical stimuli and HIF-1α activation under normoxia, some limitations must be considered. This work identifies NF-κB as a necessary mediator for HIF-1α upregulation and its transcriptional activation. Although the role of NF-κB as co-factor in HIF-1α activation is demonstrated under hypoxia, this interaction remains to be fully characterised under normoxia. Future research should further investigate the role of NF-κB as a co-factor in regulating HIF-1α transcriptional activation, and how inflammatory and hypoxia signalling establish a coordinated transcriptional programme in response to mechanical stimuli. The in vitro experimental models used cannot fully recapitulate the spatial and temporal complexity of tumour progression, nor reproduce the different mechanical cues of the TME (e.g., solid stress, fluid pressure, viscoelasticity, microarchitecture). These unexplored mechanical cues in a more physiological 3D environment may influence the mechanosensitive HIF-1α activation reported here. These results are also limited to PSCs, limiting translational capacity to other solid tumours. The interplay between mechanical stress and HIF-1α activation under hypoxia or in pro-inflammatory states remains incompletely understood and should be further explored at other primary tumour sites or highly desmoplastic pathological conditions [67]. Future studies should focus on validating these mechanisms in vivo within ECM-rich tissues to dissect the relationship between mechanical stress, normoxic HIF-1α activation, and the onset hypoxia. Understanding how stromal and immune cells respond to mechanosensitive HIF-1α activation is critical for translating these findings into therapeutic strategies.

## Conclusion

We present a model in which HIF-1α functions as a central integrator of mechanical stress and metabolic regulation, expanding beyond its canonical role as a hypoxia-sensitive factor. We demonstrate that mechanical stress activates HIF-1α under normoxia, and this HIF-1α transcriptional activation requires a coordinated transcriptional programme. Mechanistically, NF-κB is a key mediator for the mechanosensitive HIF-1α activation, also dependent on actomyosin contractility. These results propose a mechanosensitive signalling pathway for the β1 integrin/MLC-2/NF-κB/HIF-1α axis under normoxia. Pharmacological modulation by targeting GPER as upstream mechanoregulator with G-1 modulated HIF-1α and NF-κB activation, effectively reducing HIF-1α activation under normoxia.

## Electronic supplementary material

Below is the link to the electronic supplementary material.

Supplementary Material 1

## Data Availability

Data needed to evaluate the conclusions in the paper are present in the paper and the materials raw data are available on request.

## Abbreviations

αSMA: Alpha-smooth muscle actin
CAF: Cancer-associated fibroblast
DMOG: Dimethyloxalylglycine
EMT: Epithelial-mesenchymal transition
ECM: Extracellular matrix
FAK: Focal adhesion kinase
GPER: G protein-coupled oestrogen receptor
HRE: Hypoxia response element
HIF-1: Hypoxia-inducible factor 1
MLC-2: Myosin light chain 2
NF-κB: Nuclear factor kappa B
PAA: Polyacrylamide
PSC: Pancreatic stellate cells
PDAC: Pancreatic ductal adenocarcinoma
PHD: Prolyl hydroxylase
ROS: Reactive oxygen species
TIRF: Total internal reflection fluorescence
TME: Tumour microenvironment
pVHL: Von Hippel-Lindau
