# Supplementary material for: Substrate stiffness regulates HIF-1α signalling via β1-integrin/myosin-2/NF-κB axis
Source: J Transl Med. 2026 Jul 9;24:1241. doi: 10.1186/s12967-026-08553-9 (PMC13640376; doi:10.1186/s12967-026-08553-9)
Supplement: Supplementary file 1 [file 12967_2026_8553_MOESM1_ESM.docx]

**Supplementary material:**

**Title: Substrate stiffness regulates HIF-1α signalling via β1-integrin/myosin-2/NF-κB axis**

**Authors:** *Ander Bastida Urkiza*, Ernesto Cortes* ^§^, Chongguang Jin & Dariusz Lachowski*^§^*

*Co-first authors

§ Corresponding authors: [ernesto.corteslopez@uam.es](mailto:ernesto.corteslopez@uam.es), [dlachows@ing.uc3m.es](mailto:dariusz.lachowski@uc3m.es)

**Figure S1: Substrate stiffness modulates the mRNA expression of HIF-1α** **downstream genes.** Reverse transcription quantitative PCR detection of **(A)** LOX-L2 and **(B)** LOX-L3 genes in hPSCs cultured on soft (1 kPa) or stiff (25 kPa) substrates (n=3 biological replicates). Bars represent mean ± SEM.

**Figure S2: NF‑κB silencing does not alter PSC contractile phenotype in response to substrate stiffness. (A)** Widefield immunofluorescence images of hPSCs for alpha-smooth muscle actin (αSMA) cultured on soft (1 kPa) or stiff (25 kPa) substrates, transfected with NF**‑**κB siRNA, or treated with DMOG (100 µM, 96h). Control scramble siRNA (scr siRNA). Scale bar, 20 µm. **(B)** Total fluorescence intensity of αSMA per cell, as shown in (A) (n=25 cells).

**Figure S3: Pharmacological actomyosin contractility modulation affects the morphology of PSCs. (A)** Cell perimeter and **(B)** cell circularity measured as $4\pi\times\frac{[Area]}{{[Perimeter]}^{2}}$ quantified from the dataset represented in Figure 4A; (n=25 cells). **(C)** F-actin fibres extracted using CT-FIRE software (1) of PSCs treated with and without RhoA inhibitor (CT04; 2.0 µg/ml, 4 h) or myosin light chain 2 (MLC-2) inhibitor (Blebbistatin; 50 µM, 4 h). **(D)** Quantification of the number of actin fibres per cell, **(E)** mean fibre length per cell and **(F)** mean resultant length measured as $\sqrt{{(\frac{1}{N}\sum\cos\theta_{i})}^{2}+{(\frac{1}{N}\sum\sin\theta_{i})}^{2}}$, where *N* is the number of fibres and *θ* the fibre angle (n=25 cells).

**Figure S4: G-1 induces mechanosensitive inactivation of PSCs. (A)** Cell perimeter and **(B)** cell circularity measured as $4\pi\times\frac{[Area]}{{[Perimeter]}^{2}}$ quantified from the dataset represented in Figure 4C; (n=25 cells). **(C)** F-actin fibres extracted using CT-FIRE software (1) of PSCs treated with and without GPER agonist (G-1; 1 µM, 72 h). **(D)** Quantification of the number of actin fibres per cell, **(E)** mean fibre length per cell and **(F)** mean resultant length measured as $\sqrt{{(\frac{1}{N}\sum\cos\theta_{i})}^{2}+{(\frac{1}{N}\sum\sin\theta_{i})}^{2}}$, where *N* is the number of fibres and *θ* the fibre angle (n=25 cells). **(G)** Widefield immunofluorescence images of hPSCs for αSMA (top) and vimentin (bottom) cultured on soft (1 kPa) or stiff (25 kPa) substrates, treated with G-1 (1 µM, 72h). Scale bar, 20 µm. **(H)** Total fluorescence intensity of αSMA per cell, as shown in (A) (n=25 cells). **(I)** Total fluorescence intensity of vimentin per cell, as shown in (A) (n=25 cells).

**References**

1.	Bredfeldt JS, Liu Y, Pehlke CA, et al. Computational segmentation of collagen fibers from second-harmonic generation images of breast cancer. *J Biomed Opt*. 2014;19(1):16007.
